# Zagreb Declaration on Person Centered Health Professional Education

**DOI:** 10.3325/cmj.2014.55.81

**Published:** 2014-02

**Authors:** Board of the International College of Person-Centered Medicine (ICPCM)

To address the issue on Person Centered Health Professional Education, members of the Board of the International College of Person-Centered Medicine (ICPCM) and participants of the First International Congress of the ICPCM met during the Congress in Zagreb, Croatia on November 8th, 2013.

We, participants in the First International Congress of Person-centered Medicine and the Board of the International College of Person-Centered Medicine, call for action to promote person-centered health for all through transformative educational concepts, programs and actions.

Person Centered Medicine seeks to place the whole person in context at the center and as the goal of clinical practice and public health. It articulates science and humanism to achieve health care actions that are ethical, holistic, culturally sensitive, relationally attentive, individualized and seek common ground for joint diagnostic understanding and shared decision-making for prevention, treatment, rehabilitation and health promotion. It also endeavors for people-centered development of health services and public health policies, all supported by person-centered education, training and research.

In order to advance person-centered medicine and health through educational efforts, we derive strength, inspiration and guidance from the many international bodies that regularly participate in our discussions, among them the World Federation for Medical Education, the International Association of Medical Colleges, and academic institutions from across the world. We also draw information and documentation from the proceedings of relevant events such as the the Geneva Conferences and International Congresses of Person-centered Medicine as well as from pertinent publications such as the International Journal of Person Centered Medicine.

Person-centered educational efforts need to be attentive to the different aspects and levels of the educational domain, including conceptual bases, institutional organization and culture, selection and development of health professional students, instructors and mentors, interactional mechanisms among students, teachers, patients and families, educational settings, curriculum development and evaluation, educational methods, and broader issues on education, health and society.

## Recommendations

We therefore propose the following educational and related actions:

1. Enhancement of the conceptual bases of professional training and public education by grounding them in ethics and human rights, knowledge of the bio-psycho-socio-cultural determinants of ill health and positive health, attention to personal values and preferences, and the flourishing of well-being and life projects.

2. Analysis and optimization of the organization and culture of educational institutions to be supportive of person-centered health for and with students, faculty and administrative colleagues.

3. Selection of students who are suited to the goals and responsibilities required of health professionals and commitment to their personal development in addition to their broad technical training.

4. Selection of instructors and mentors who are suited to their educational responsibilities and commitment to their continuing professional and personal development.

5. Facilitation of dynamic interactions among students, instructors, patients and families by ensuring opportunities for dialogue at all levels, promoting inter-disciplinary training, and engaging patients as instructors.

6. Promoting curricular developments that have person-centered guiding principles, that include curricular experiments and mechanisms for their evaluation, that consider a balance between concentrated blocs and longitudinal integration for the achievement of educational objectives, and that incorporate the continuum of health promotion, prevention, treatment, rehabilitation and palliative care.

7. Exploring the design, implementation and evaluation of person-centered educational methods such as experiential and reflexive learning, communicational exercises, training groups with patients and families, shadowing mentors, employing video recordings for feedback purposes, community visits and practice, utilization of narratives, practice of artistic activities, motivational interviewing, establishing common ground among clinicians, patients and families for diagnosis and care, articulation of competence- and performance-training, and ability to work with patients from different cultures and social conditions in a respectful and collaborative manner.

8. Promoting health in individual patients and in society by active education of patients, addressing broad issues on determinants of health and the impact of cultural, religious and social factors on health and health care, identifying and reducing health hazards in the environment and society, and being all involved in shaping sound health policies.

The International College of Person-centered Medicine is committed to the above listed recommendations and calls on governmental, intergovernmental and non-governmental organizations to collaborate on the advancement of these educational efforts toward the promotion of person-centered medicine and health.

